# Antigen-responsive CD4^+^ T cell clones contribute to the HIV-1 latent reservoir

**DOI:** 10.1084/jem.20200051

**Published:** 2020-04-20

**Authors:** Pilar Mendoza, Julia R. Jackson, Thiago Y. Oliveira, Christian Gaebler, Victor Ramos, Marina Caskey, Mila Jankovic, Michel C. Nussenzweig, Lillian B. Cohn

**Affiliations:** 1 Laboratory of Molecular Immunology, The Rockefeller University, New York, NY; 2 The Chan Zuckerberg Biohub, San Francisco, CA; 3 Howard Hughes Medical Institute, The Rockefeller University, New York, NY; 4 Department of Medicine, University of California, San Francisco, San Francisco, CA

## Abstract

Antiretroviral therapy suppresses but does not cure HIV-1 infection due to the existence of a long-lived reservoir of latently infected cells. The reservoir has an estimated half-life of 44 mo and is largely composed of clones of infected CD4^+^ T cells. The long half-life appears to result in part from expansion and contraction of infected CD4^+^ T cell clones. However, the mechanisms that govern this process are poorly understood. To determine whether the clones might result from and be maintained by exposure to antigen, we measured responses of reservoir cells to a small subset of antigens from viruses that produce chronic or recurrent infections. Despite the limited panel of test antigens, clones of antigen-responsive CD4^+^ T cells containing defective or intact latent proviruses were found in seven of eight individuals studied. Thus, chronic or repeated exposure to antigen may contribute to the longevity of the HIV-1 reservoir by stimulating the clonal expansion of latently infected CD4^+^ T cells.

## Teaser

Mendoza et al. identify clones of HIV-1 defective or intact latent proviruses within antigen-responsive CD4^+^ T cells. This study suggests that exposure to chronic or recurrent antigens may contribute to reservoir persistence by stimulating the clonal expansion of latently infected CD4^+^ T cells.

## Introduction

After integration into the host genome, HIV-1 transcription usually leads to new virus production and cell death. However, HIV-1 can also become latent in a small number of infected CD4^+^ T cells, and these cells constitute a latent reservoir that is the principle barrier to HIV-1 cure (Bruner and Cohn, 2019). The latent reservoir has a long half-life of ∼44 mo (Crooks et al., 2015; Siliciano et al., 2003) and persists in memory CD4^+^ T cells, including some that are HIV-1, CMV, and influenza specific (Douek et al., 2002; Jones et al., 2012; Demoustier et al., 2002; Hey-Nguyen et al., 2019).

A significant fraction of the circulating latent reservoir is composed of expanded clones of CD4^+^ T cells containing replication-competent proviruses (≥50%; Bui et al., 2017; Hosmane et al., 2017; Lorenzi et al., 2016; Simonetti et al., 2016; Reeves et al., 2018; Lu et al., 2018; Cohen et al., 2018). Although the origin of the clones and the mechanisms that govern their expansion is not known, longitudinal analysis indicates that they are dynamic and change in size over time in individuals who maintain viral suppression on antiretroviral therapy (ART; Wang et al., 2018; Cohn et al., 2015; Wagner et al., 2014).This dynamic may partially account for the longevity of the reservoir (Bruner and Cohn, 2019). Thus, understanding the basis for latently infected T cell clonal expansion is important for learning how to control and potentially eliminate the reservoir.

HIV-1 proviral DNA is enriched in HIV-1–, CMV-, and influenza-responsive T cells obtained from ART-suppressed individuals, but whether or how this might be related to clonal expansion of T cells harboring latent viruses that remain replication competent has not been examined (Hey-Nguyen et al., 2019; Kristoff et al., 2019; Henrich et al., 2017; Douek et al., 2002; Demoustier et al., 2002; Jones et al., 2012). Here, we report that CD4^+^ T cells containing clones of replication-competent viruses respond to antigenic stimulation with peptides derived from viruses that cause chronic or recurrent infections.

## Results and discussion

**Figure 1.**
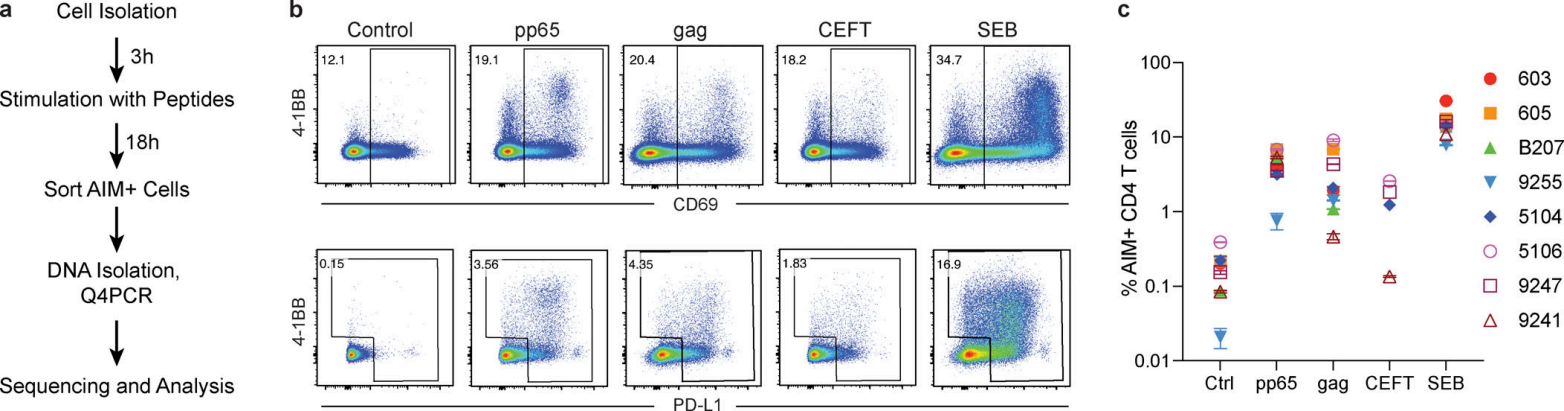
**AIM assay.** **(a)** Experimental overview. PBMCs were depleted of CD8^+^ T cells and rested for 3 h before stimulation for 18 h with peptide pools. Cells were then purified based on expression of AIMs (CD69, PD-L1, and 4-1BB; Fig. S1). DNA was isolated from sorted cells, Q4PCR was performed, and sequenced viruses were assembled and analyzed. **(b)** Representative example of cell purification by AIM after stimulation with CMV-pp65, HIV-gag, CEFT (from four of eight donors), or SEB by gating on CD69-positive cells followed by gating on PD-L1–positive or 4-1BB–positive cells. Control CD4^+^ T cells cultured with MOG were purified on the basis of CD4 expression alone. Each AIM assay staining was performed twice on each donor (*n* = 8). Numbers represent the percentage of total CD4^+^ T cells within each gate. **(c)** Frequency of AIM^+^ cells across all donors. Cells were processed and sorted as in panel b.

**Figure S1.**
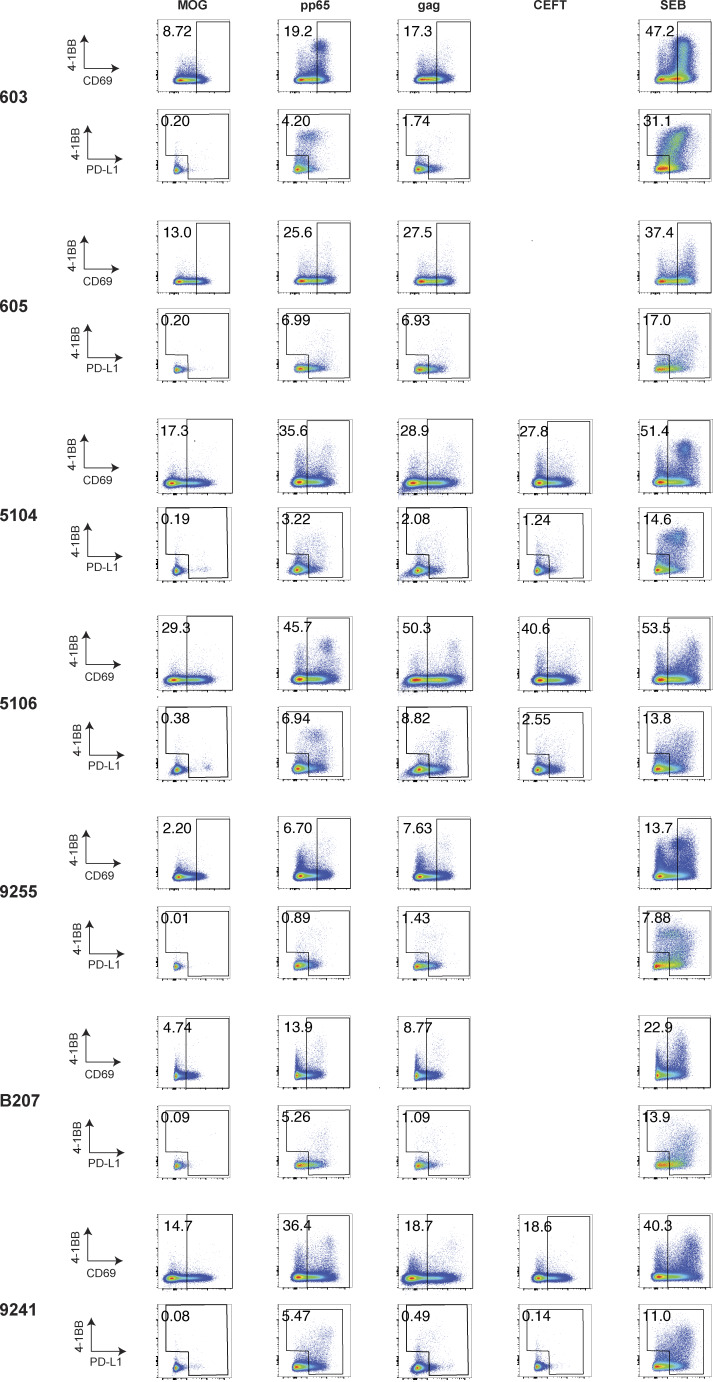
**AIM flow cytometry plots.** CD4^+^ T cells were stimulated for 18 h with peptide pools from MOG (control), CMV-pp65, HIV-gag, CEFT, or SEB before staining. CD4^+^ T cells were gated on CD69-positive cells, followed by gating on PD-L1–positive and/or 4-1BB–positive cells. Each AIM assay staining was performed twice on each donor. Numbers represent the percentage of total CD4^+^ T cells within each gate.

To test the hypothesis that expanded clones harboring latent proviruses respond to foreign antigens, we exposed CD4^+^ T cells from ART-suppressed individuals (Mendoza et al., 2018; Cohen et al., 2018; NCT03571204; Table S1 and Table S2) to overlapping peptide pools from common viral and bacterial antigens including HIV group specific antigen (HIV-gag), CMV phosphoprotein 65 (CMV-pp65), or pooled peptides from CMV, EBV, influenza, and tetanus toxin (CEFT). Some of these antigens have been shown to induce HIV-RNA transcription in vivo after vaccination (Stanley et al., 1996; van Sighem et al., 2008). Staphylococcal enterotoxin B (SEB) and a self-protein, myelin oligodendrocyte glycoprotein (MOG), served as positive and negative controls, respectively, for T cell activation. After overnight culture with HIV-gag, CMV-pp65, CEFT, or SEB, activated CD4^+^ T cells from eight donors were purified by cell sorting based on expression of two or more activation-induced markers (AIMs; CD69 and PD-L1 or 4-1BB; Dan et al., 2016; Reiss et al., 2017; Havenar-Daughton et al., 2016; Fig. 1, a and b; and Fig. S1). Total live CD4^+^ T cells were sorted from parallel cultures stimulated with MOG to serve as unfractionated controls that were subjected to the same processing conditions. As expected, there was little detectable response to the MOG self-antigen peptide pool, and all donors showed high-level responses to SEB. In addition, responses to HIV-gag, CMV-pp65, and CEFT varied in magnitude among individuals (Figs. 1 c and S1).

**Figure 2.**
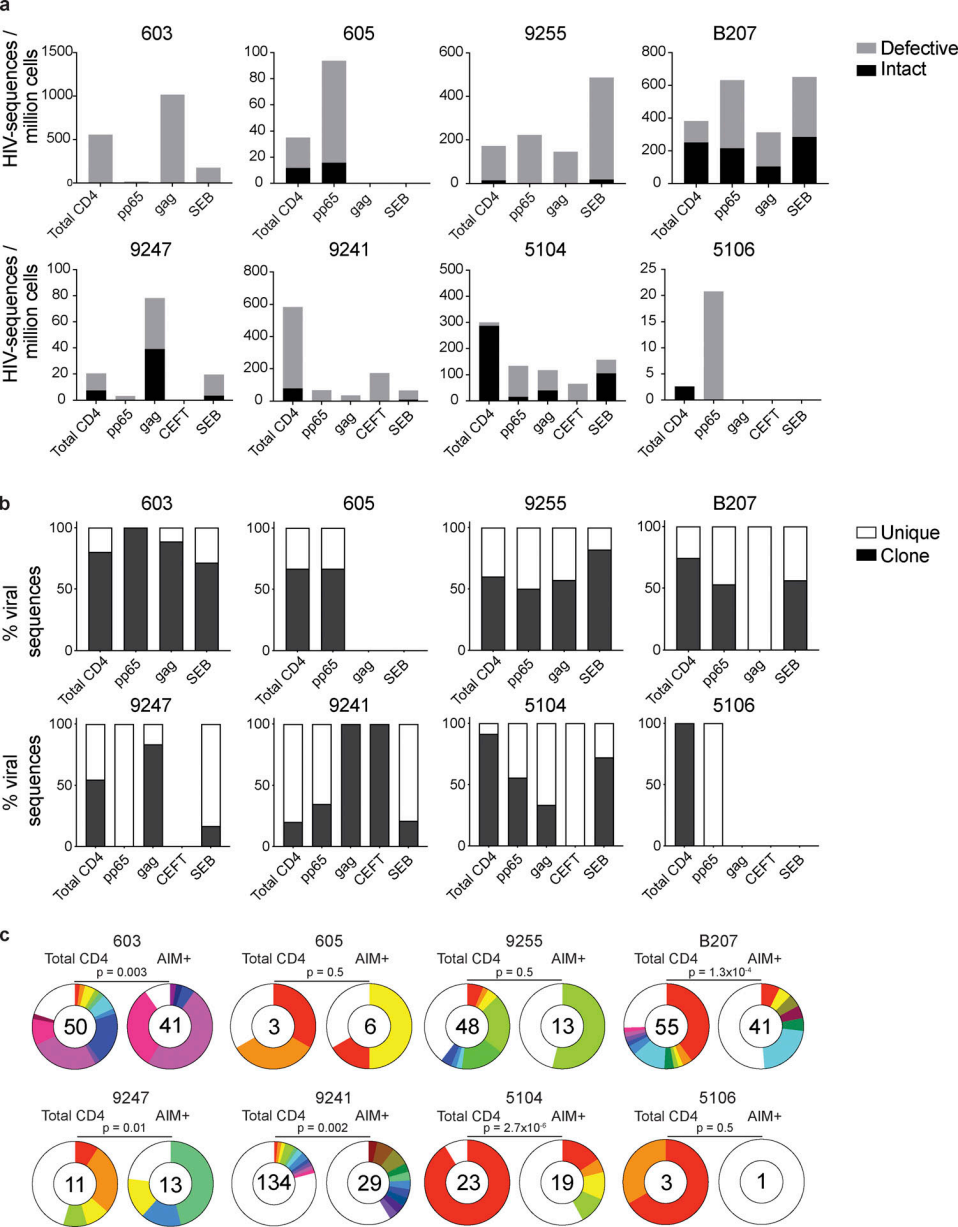
**Defective and intact proviral analysis from antigen-responsive cells.** **(a)** Frequency of intact (black) and defective (gray) viruses characterized by Q4PCR per million AIM^+^ CD4^+^ T cells from eight donors. **(b)** Frequency of virus sequences identified only once (unique) or more than once (clonal) across baseline and all sorted populations from each donor. **(c)** Clonal distribution of Q4PCR-derived HIV-1 sequences from total CD4^+^ T cells (MOG control) and all antigen-reactive AIM^+^ CD4^+^ T cells combined, from eight donors. Numbers in the inner circles indicate the total number of HIV-1 sequences analyzed. White represents sequences identified only once across all conditions (unique), and colored slices represent sequences identified more than once (clones) across baseline and all sorted populations from each donor. The size of each pie slice is proportional to the size of the clone. P values denote a significant change in overall clonal distribution using a two-sided Fisher’s exact test. Total DNA extracted from sorted populations was diluted across individual PCR reactions (ranging from 384 to 6,144 reactions) based on gag dilution (see Table S3).

To determine whether CD4^+^ T cells harboring HIV-1 proviruses are enriched among antigen- or SEB-responsive cells compared with the MOG control, integrated proviruses were enumerated and further characterized as intact or defective by combining quantitative PCR (qPCR) and next-generation sequencing (quadraplex qPCR [Q4PCR]; Gaebler et al., 2019; Fig. 2 a). The combination of PCR and sequencing methods enables initial selection of likely-intact viruses by PCR, and these are subsequently confirmed as intact or defective by near-full-length (NFL) proviral amplification and sequencing (Gaebler et al., 2019). As expected, we observed variation in the ratio of intact to defective proviruses between individuals (Ho et al., 2013; Fig. 2 a).

The overall frequency of intact and defective proviruses contained within antigen-responsive cells varied substantially among individuals. For example, proviruses were nearly absent from CMV-pp65–specific CD4^+^ T cells in participant 603 and absent from HIV-gag–specific CD4^+^ T cells in participants 605 or and 5106. In contrast, 1 in 1,000 HIV-gag–responsive cells harbored a provirus in 603 (Fig. 2 and Table S3). Individuals 603 and 9247 showed proviral enrichment in CD4^+^ T cells responding to gag of 2- and 4-fold, respectively, whereas in 605 and 5106, proviruses were 3- and 10-fold enriched in pp65-responsive CD4^+^ T cells (Fig. 2 a). Finally, antigen-responsive cells were relatively depleted of integrated proviruses in participants 9241 and 5104 (Fig. 2 a). We conclude that HIV-1 proviruses can persist in CD4^+^ T cells that respond to CMV-pp65 and HIV-gag antigen with strong variation among infected individuals.

**Figure S2.**
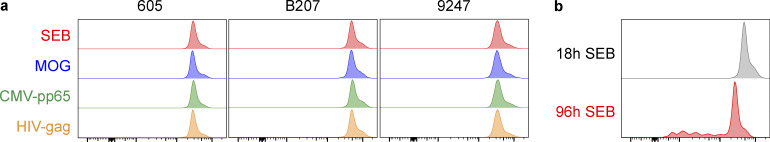
**CFSE proliferation assay after 18-h stimulation.** **(a)** CFSE-labeled CD4^+^ T cells from participants 605, B207, and 9247 were stimulated with peptide pools from MOG (control), CMV-pp65, HIV-gag, or SEB before staining. Histograms show CFSE labeling of CD4^+^ T cells after 18 h of stimulation. **(b)** Representative histograms of CFSE labeling within CD4^+^ T cells for participant B207 after 18 and 96 h of stimulation with SEB, respectively. Each AIM assay staining was performed twice on each donor.

We analyzed all HIV-1 sequences across all groups to determine the fraction of viral sequences contributing to expanded clones of CD4^+^ T cells. Sequences found more than once were classified as derived from clones, while unique sequences were those identified only once. As expected (Bui et al., 2017; Lorenzi et al., 2016; Simonetti et al., 2016; Wang et al., 2018; Cohn et al., 2015, 2018; Maldarelli et al., 2014; Cohen et al., 2018; Lu et al., 2018), clones of viral sequences were identified in all participants (Fig. 2 b). The clonal distribution of HIV-1 sequences in antigen-responsive (HIV-1-gag, CMV-pp65, and CEFT) cells was significantly different from the MOG control in five of the six individuals from whom we were able to obtain >10 sequences (Fig. 2 c). The clonality was not due to T cell division in vitro, as there was no measurable T cell division in 18 h under our culture conditions (Fig. S2). We conclude that cells responding to HIV-gag and CMV-pp65 peptides contain clonal proviruses.

**Figure 3.**
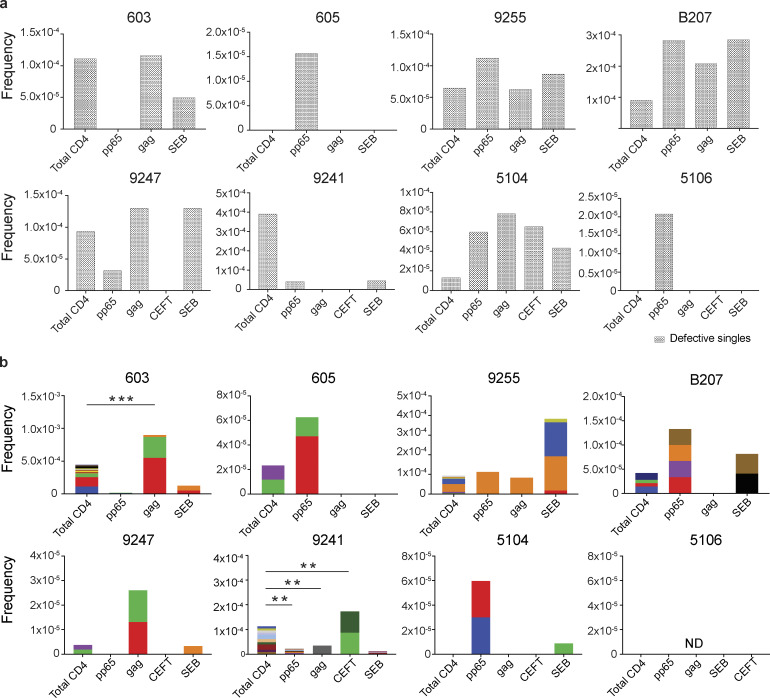
**Defective proviral analysis from antigen-responsive cells.** **(a)** Frequency of unique defective proviruses isolated from AIM^+^ cells, calculated by dividing number of sequences by total number of CD4^+^ T cells analyzed. **(b)** Frequency of clonal defective proviruses isolated from AIM^+^ cells, calculated by dividing number of clonal sequences by total number of CD4^+^ T cells analyzed. Each color represents a unique clone. In each donor, clones identified in more than one fraction of cells are represented by the same color. Asterisks denote a significant change in overall clonal distribution (**, P ≤ 0.01; ***, P ≤ 0.001, two-sided Fisher’s exact test). Total DNA extracted from sorted populations was diluted across individual PCR reactions (ranging from 384 to 6,144 reactions) based on gag dilution (see Table S3).

To determine how unique and clonal proviruses contributed to the overall enrichment observed among total HIV-1 sequences, we analyzed the sequences obtained from defective proviruses. Unique sequences were enriched in CMV-pp65–responsive cells from participants 605, 9255, 5104, and 5106 compared with total live CD4^+^ T cells in the MOG control (Fig. 3 a). There was also decreased representation of unique sequences in response to some antigens in 9247 and 9241 (Fig. 3 a). Otherwise, no distinct pattern emerged from the analysis of defective single viruses, and there was no significant antigen-dependent enrichment among unique sequences between the other individuals.

Conversely, compared with total live CD4^+^ T cells in the MOG control, seven of the eight individuals tested showed clones of identical defective proviruses in HIV-gag–, CMV-pp65–, or CEFT-responsive CD4^+^ T cells (Fig. 3 b). The overall enrichment of defective clonal sequences among antigen-responsive cells was often attributable to one or more specific clones. For example, in individuals 603 and 9247, the relative enrichment of defective proviruses corresponds to two clones isolated from HIV-gag–responsive CD4^+^ T cells, whereas two defective clones in 605 and 5104 and four defective clones in B207 account for the HIV-1 enrichment in CMV-pp65–responsive CD4^+^ T cells (Fig. 3 b). In addition, participant 9241 showed an enrichment of two proviral clones in CEFT-responsive cells (Fig. 3 b). Because defective viruses cannot infect multiple individual cells, identical defective viruses can arise due to cellular proliferation. We conclude that expanded CD4^+^ T cell clones harboring defective HIV-1 proviruses are responsive to peptides derived from HIV-gag, CMV-pp65, and CEFT antigens.

SEB is a superantigen that stimulates T cells expressing only a subset of T cell receptors (Llewelyn et al., 2006). Consistent with this limited specificity, we found that four of the eight individuals tested (9255, B207, 9247, and 5104) had expanded or novel clones of defective proviruses in SEB-reactive CD4^+^ T cells, compared with the MOG control (Fig. 3 b).

**Figure 4.**
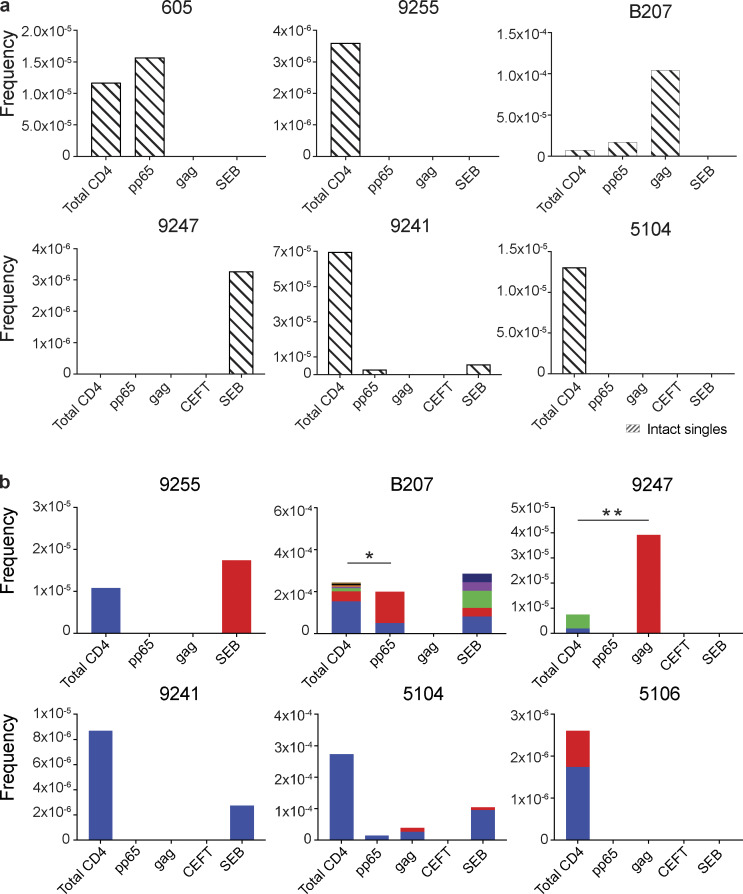
**Intact proviral analysis from antigen-responsive cells.** **(a)** Frequency of unique intact proviruses isolated from AIM^+^ cells. **(b)** Frequency of clonal intact proviruses isolated from AIM^+^ cells. Each color represents a unique clone. In each donor, clones identified in more than one fraction of cells are represented by the same color. Asterisks denote a significant change in overall clonal distribution (*, P ≤ 0.05; **, P ≤ 0.01, two-sided Fisher’s exact test). Total DNA extracted from sorted populations was diluted across individual PCR reactions (ranging from 384 to 6,144 reactions) based on gag dilution (see Table S3).

Similar analysis was also performed using intact proviruses, which, as expected, were present in much smaller numbers than defective proviruses (Fig. 4; Ho et al., 2013; Bruner et al., 2016; Hiener et al., 2017; Lu et al., 2018; Gaebler et al., 2019). Unique proviruses were enriched in HIV-gag–specific CD4^+^ T cells in participant B207, while in 605, they were found in CMV-pp65–responsive cells (Fig. 4 a).

Antigen- or SEB-responsive CD4^+^ T cell clones harboring intact proviruses were identified in three and four of the eight participants, respectively (Fig. 4 b). In B207 and 9247, we found clones of CD4^+^ T cells responding to CMV-pp65 and HIV-gag, respectively. In 9247, this activity was due to a single intact clone of identical proviruses. Although intact proviral clones were found in total CD4^+^ T cells in the MOG control in participants 9255, 9241, 5104, and 5106, they were nearly entirely absent from antigen-responsive cells (Fig. 4 b). However, the absence of such sequences might be due to the limited number of cells and panel of antigens assayed. We conclude that cells harboring intact proviruses can respond to peptides from HIV-1 and CMV antigens.

**Figure 5.**
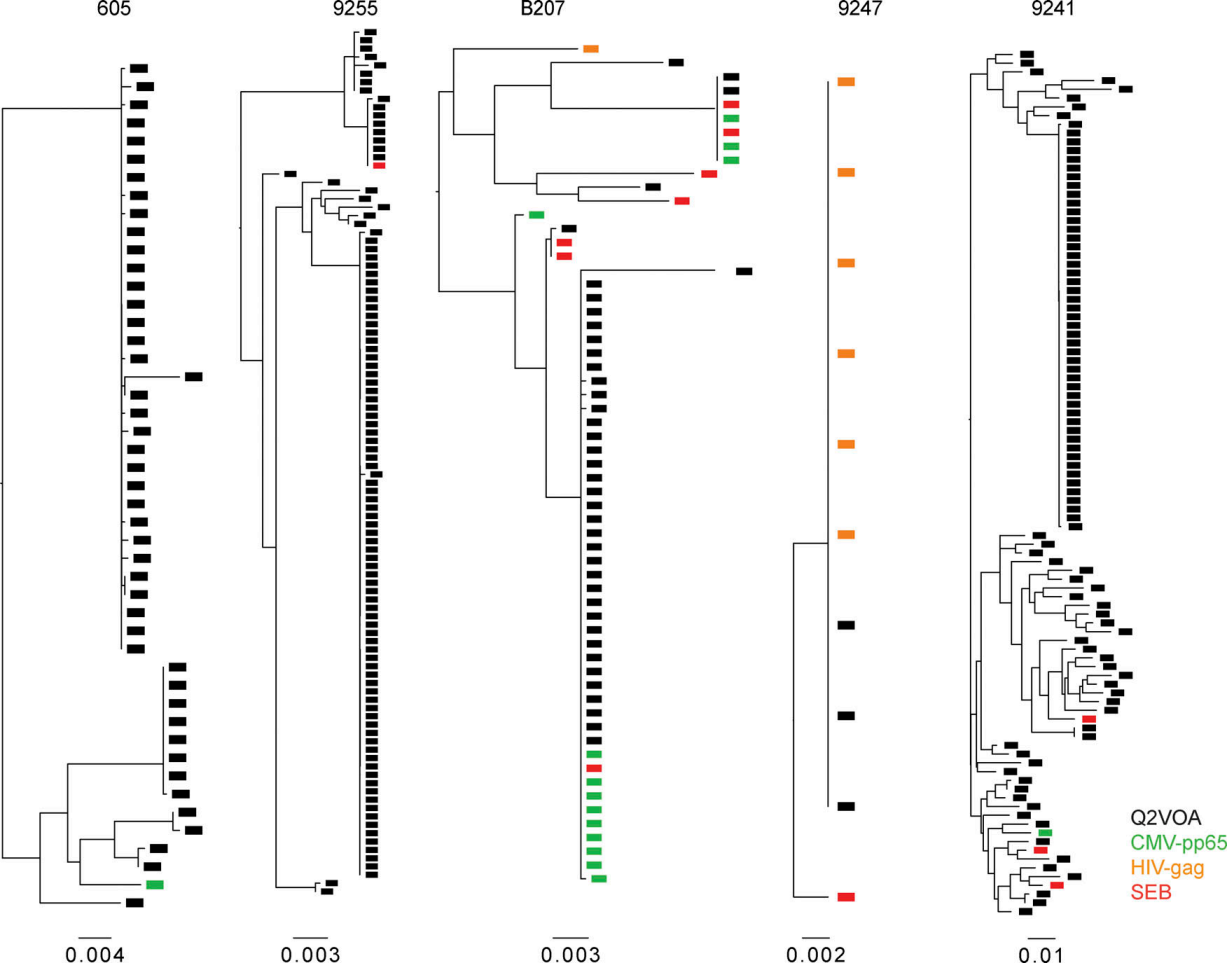
**Phylogenetic tree of *env* sequences.** Maximum likelihood phylogenetic trees of *env* sequences from intact viruses obtained from AIM^+^ cells (green, CMV-pp65; orange, HIV-gag; red, SEB) and Q^2^VOA single-cell virus outgrowth cultures (black).

To determine whether the intact sequences isolated from antigen-responsive cells are replication competent, we compared the sequences obtained from antigen- or SEB-responsive cells to those obtained from single-cell viral outgrowth cultures (quantitative and qualitative viral outgrowth assay [Q^2^VOA]) from five of the individuals for whom such data were available (Cohn et al., 2018; Mendoza et al., 2018). We found identical matches between outgrowth cultures and proviral sequences isolated from either CMV-pp65– or HIV-gag–responsive cells in B207 and 9247 (Fig. 5). Additionally, in participant B207, the intact viruses obtained from the largest CMV-pp65–responsive clone were identical to the clone isolated by latency capture (Cohn et al., 2018). Each member of this clone of CD4^+^ T cells expressed identical T cell receptor β and α chains (TRBV7-8 and TRAV-21; Cohn et al., 2018). Identical matches for proviruses harbored in SEB-reactive CD4^+^ T cells were also found in 9255 and B207. Finally, closely related sequences were found in the remaining individuals (Fig. 5). We conclude that clones of CD4^+^ T cells responsive to HIV-gag, CMV-pp65, and SEB harbor replication-competent viruses.

Understanding the mechanism of latent reservoir persistence is critical to developing methods for HIV-1 eradication or functional cure. We have used a limited number of antigens to test the idea that CD4^+^ T cells harboring identical latent proviruses can undergo clonal expansion in response to antigenic stimulation. Our results suggest a mechanism for viral persistence whereby clones of infected cells harboring latent proviruses are stimulated to divide during immune responses.

A number of ideas have been proposed to explain HIV-1 persistence. These include low-level ongoing HIV-1 replication in sanctuary sites, where drug concentrations are insufficient to block virus replication; and infected T cell longevity (Hatano et al., 2013; Sengupta and Siliciano, 2018). Cell division was not initially considered as a mechanism of proviral persistence because the signals that induce T cell division, such as NF-κB, also tend to reactivate latent proviruses leading to cell death (Siliciano and Greene, 2011). However, several lines of evidence support the idea that CD4^+^ T cells harboring latent proviruses can undergo clonal expansion. Initial indirect support for clonal expansion came from longitudinal human studies showing that ART suppressed individuals produce closely related viruses at low levels over time (Bailey et al., 2006; Tobin et al., 2005). Direct evidence for the existence of expanded clones was obtained from independent cultures of latent CD4^+^ T cells in limiting dilution viral outgrowth assays, suggesting that ≥50% of the circulating reservoir consists of expanded clones (Lorenzi et al., 2016; Hosmane et al., 2017; Bui et al., 2017; Simonetti et al., 2016; Wiegand et al., 2017). In addition, proviral integration site sequencing revealed collections of cells that share identical proviral integration sites (Cohn et al., 2015; Wagner et al., 2014; Maldarelli et al., 2014; McManus et al., 2019). Furthermore, in vitro studies showed that cell division and latency are not mutually exclusive (Wang et al., 2018; Hosmane et al., 2017; Pinzone et al., 2019). Finally, isolation of productively infected cells and paired TCR and full-length viral sequencing (Cohn et al., 2018) and paired full-length viral sequencing and integration site analysis (Einkauf et al., 2019) provided definitive evidence for the existence of expanded clones of CD4^+^ T cells harboring identical intact latent proviruses.

Despite compelling evidence for CD4^+^ T cell proliferation as a mechanism for HIV-1 persistence, how latently infected cells divide without succumbing to cytopathic viral reactivation remains unknown. Possible explanations include expression of genes that favor survival by suppressing viral transcription (Cohn et al., 2018), viral integration in transcriptionally inactive regions of the genome (Craigie and Bushman, 2012), and expression of antiapoptotic proteins such as BIRC5 (Kuo et al., 2018).

Although there is general agreement that a large fraction of the HIV-1 reservoir comprises clones of CD4^+^ T cells that wax and wane (Wang et al., 2018; Lorenzi et al., 2016; Cohen et al., 2018), far less is understood about what triggers clonal expansion and maintains reservoir longevity. It has been proposed that proviral integration in the vicinity of genes that control cell division, such as cancer-associated genes, promotes cell growth (Maldarelli et al., 2014; Wagner et al., 2014). However, HIV-1 is preferentially integrated into highly transcribed genes (Schröder et al., 2002) that include many cancer-associated genes. Thus, it has been difficult to definitively determine whether or how integration in the vicinity of cancer-related genes contributes to HIV-1 persistence (Cohn et al., 2015; Einkauf et al., 2019). Moreover, unlike transforming retroviruses that integrate into cancer genes and cause unrestricted cell growth, HIV-1 is not known to cause T cell cancers by integration.

Our data may provide mechanistic insights into the finding that immunization with tetanus toxoid or influenza can in some, but not all cases, produce increases in HIV-1 RNA in plasma (Christensen-Quick et al., 2018; Stanley et al., 1996). Individuals with vaccine-responsive T cell clones that harbor intact latent viruses would be expected to produce variable amounts of virus after vaccination. The data are also consistent with the report that viral load blips are more frequent during winter months, when there is a higher incidence of seasonal viral infections (van Sighem et al., 2008).

Despite the use of a very limited number of test antigens, our work suggests that expanded clones of CD4^+^ T cells containing replication-competent proviruses respond to pathogens. Their intermittent exposure to these and other antigens found in the virome and microbiome may account for the reported waxing and waning of individual clones of latently infected CD4^+^ T cells and their persistence over time. Finally, barrier tissues that are chronically exposed to antigen, such as the skin or gut, may represent reservoir sites that contribute to persistence of the reservoir by supporting ongoing expansion and contraction of CD4^+^ T cell clones harboring latent proviruses.

## Materials and methods

### Study design and participants

All study participants were recruited at the Rockefeller University Hospital, New York, NY, and the University Hospital Cologne, Cologne, Germany (Mendoza et al., 2018; Cohen et al., 2018; and https://clinicaltrials.gov/ct2/show/NCT03571204). All participants provided written informed consent before participation in the studies. The studies were conducted in accordance with Good Clinical Practice. The protocols were approved by the institutional review boards at the Rockefeller University and the University of Cologne. Peripheral blood mononuclear cells (PBMCs) were isolated from leukapheresis by Ficoll separation and frozen in aliquots. All participants were confirmed to be aviremic at the time of sample collection. The samples used in the study described here were collected before any investigational therapeutic intervention.

### CD4^+^ T cell isolation

Total CD4^+^ T cells from baseline leukapheresis were isolated from cryopreserved PBMCs by manual magnetic labeling and negative selection using the CD4^+^ T Cell Isolation Kit (Miltenyi Biotec).

### AIM assay

PBMCs were thawed and washed, and CD8^+^ T cells were depleted using human CD8 MicroBeads (Miltenyi Biotec, 130-045-201). CD8^+^ T cell–depleted PBMCs were cultured in 24-well plates at a concentration of 10 × 10^6^ cells/ml in RPMI 1640 supplemented with Hepes, penicillin/streptomycin, and 10% human serum (Sigma-Aldrich). Cells were rested for 3 h and then stimulated with 0.5 µg/ml of HIV-1 consensus B Gag pool, CMV peptide pool, and CEFT peptide pool (PM-HIV-CONB, PM-PP65-2, PM-CEFT-MHC-II, all from JPT Peptide Technologies) for 18 h at 37°C, 5% CO_2_. A MOG peptide pool (0.5 µg/ml, PM-MOG, JPT Peptide Technologies) served as negative control. SEB-stimulated cells (0.5 µg/ml, Toxin Technology, BT202) served as positive control. Cells were stained for viability with Aquavivid (Life Technologies) at 1/200 in PBS for 20 min, 4°C; incubated with human FcR Block (Miltenyi Biotec, 130-059-901) at 1/70 in PBS-FBS 1% for 10 min, 4°C; and stained with antibodies to surface markers for 30 min, 4°C with Brilliant Violet Buffer (BD Biosciences, 566349) at 1/4 in PBS-FBS 1% (see Table S4 for antibody staining panel).

### Cell sorting

AIM^+^ cells were gated on live, single cells, CD14^−^CD19^−^CD8^−^CD4^+^, and were sorted based on expression of activation markers CD69 and PD-L1 and/or 4-1BB, as previously described (Dan et al., 2016; Reiss et al., 2017; Havenar-Daughton et al., 2016; Niessl et al., 2020). This combination of markers was chosen because of its specificity for activated memory CD4^+^ T cells with limited background binding by other T cell subsets. MOG- and SEB-treated cells served as negative and positive controls, respectively, to set gates for sorting. MOG-stimulated cells were sorted based on CD14^−^CD19^−^CD8^−^CD4^+^ expression. CD69 single-positive cells and AIM^−^ cells (CD69^−^, 4-1BB^−^, PD-L1^−^) were also sorted as controls. Cells were sorted on a FACS Aria II flow cytometer (BD Biosciences) into tubes containing RPMI 1640 supplemented with 10% FBS, Hepes and penicillin/streptomycin. Cells were pelleted, and cell pellets were flash frozen on dry ice and subsequently processed for DNA extraction as described below.

### Data analysis

Flow cytometric data were analyzed using FlowJo version 10.6.0 for Mac. R programming language was used to apply Fisher’s exact test to evaluate whether there was a statistically significant change in the overall distribution of the clones between the Q4PCR-derived HIV-1 sequences from total CD4^+^ T cells (MOG control) and all antigen-reactive AIM^+^ CD4^+^ T cells.

### DNA isolation and quantification

Genomic DNA from sorted CD4^+^ T cells was isolated using phenol-chloroform. Briefly, CD4^+^ T cells were lysed in proteinase K buffer (100 mM Tris, pH 8, 0.2% SDS, 200 mM NaCl, and 5 mM EDTA) and 20 mg/ml proteinase K at 56°C for 12 h, followed by genomic DNA extraction with phenol/chloroform/isoamyl alcohol (Invitrogen), nonfluorescent pellet paint (Millipore, 70748), and ethanol precipitation. DNA concentration was measured by Qubit High Sensitivity Kit (Thermo Fisher Scientific).

### Limiting dilution *gag* qPCR

To get a measurement of cells containing gag^+^ proviruses, genomic DNA was serially diluted and assayed by gag qPCR (concentrations ranged from 600 to 12.5 CD4^+^ T cells per well). Based on the Poisson distribution, when <30% of qPCR reactions are positive, each positive PCR reaction has a >80% probability of containing a single copy of HIV-1 DNA (Lu et al., 2018). DNA was assayed in a minimum of 16 reactions per concentration in a 384-well plate format using the Applied Biosystem QuantStudio 6 Flex Real-Time PCR System. HIV-1–specific primers and a probe targeting a conserved region in *gag* were used in a limiting dilution qPCR reaction (forward primer, 5′-ATG​TTT​TCA​GCA​TTA​TCA​GAA​GGA-3′; internal probe, 5′-/6-FAM/CCACCCCAC/ZEN/AAGATTTAAACACCATGCTAA-3′/IABkFQ; reverse primer, 5′-TGC​TTG​ATG​TCC​CCC​CAC​T-3′; Integrated DNA Technologies; Palmer et al., 2003).

Each qPCR reaction was performed in a 10-µl total reaction volume containing 5 µl of TaqMan Universal PCR Master Mix containing Rox (4304437; Applied Biosystems), 1 µl of diluted genomic DNA, nuclease-free water, and 337.5 nM of forward and reverse primers with 93.75 nm of *gag* internal probe. *gag* qPCR conditions were 94°C for 10 min, 50 cycles of 94°C for 15 s, and 60°C for 60 s.

### NFL HIV-1 PCR (NFL1)

We used a two-step nested PCR approach to amplify NFL HIV-1 genomes. All reactions were performed in a 20-µl reaction volume using Platinum Taq High Fidelity polymerase (Thermo Fisher Scientific). The outer PCR reaction (NFL1) was performed on genomic DNA at a single-copy dilution (previously determined by *gag* limiting dilution) using outer PCR primers BLOuterF (5′-AAA​TCT​CTA​GCA​GTG​GCG​CCC​GAA​CAG-3′) and BLOuterR (5′-TGA​GGG​ATC​TCT​AGT​TAC​CAG​AGT​C-3′). Touchdown cycling conditions were 94°C for 2 min and then 94°C for 30 s, 64°C for 30 s, and 68°C for 10 min for three cycles; 94°C for 30 s, 61°C for 30 s, and 68°C for 10 min for three cycles; 94°C for 30 s, 58°C for 30 s, and 68°C for 10 min for three cycles; 94°C for 30 s, 55°C for 30 s, and 68°C for 10 min for 41 cycles; and then 68°C for 10 min (Li et al., 2007; Ho et al., 2013).

### Q4PCR

Q4PCR was performed as previously described (Gaebler et al., 2019). Briefly, undiluted 1-µl aliquots of the NFL1 PCR product were subjected to a Q4PCR reaction using a combination of four primer/probe sets that target conserved regions in the HIV-1 genome. Each primer/probe set consists of a forward and reverse primer pair as well as a fluorescently labeled internal hydrolysis probe as follows: PS forward, 5′-TCT​CTC​GAC​GCA​GGA​CTC-3′; reverse, 5′-TCT​AGC​CTC​CGC​TAG​TCA​AA-3′; probe, 5′-/Cy5/TTTGGCGTA/TAO/CTCACCAGTCGCC-3′/IAbRQSp (Integrated DNA Technologies; Bruner and Siliciano, 2016); *env* forward, 5′-AGT​GGT​GCA​GAG​AGA​AAA​AAG​AGC-3′; reverse, 5′-GTC​TGG​CCT​GTA​CCG​TCA​GC-3′; probe, 5′-/VIC/CCTTGGGTTCTTGGGA-3′/MGB (Thermo Fisher Scientific; Bruner et al., 2016); *gag* forward, 5′-ATG​TTT​TCA​GCA​TTA​TCA​GAA​GGA-3′; reverse, 5′-TGC​TTG​ATG​TCC​CCC​CAC​T-3′; probe, 5′-/6-FAM/CCACCCCAC/ZEN/AAGATTTAAACACCATGCTAA-3′/IABkFQ (Integrated DNA Technologies; Palmer et al., 2003); and *pol* forward, 5′-GCA​CTT​TAA​ATT​TTC​CCA​TTA​GTC​CTA-3′; reverse, 5′-CAA​ATT​TCT​ACT​AAT​GCT​TTT​ATT​TTT​TC-3′; probe, 5′-/NED/AAGCCAGGAATGGATGGCC-3′/MGB (Thermo Fisher Scientific; Schmid et al., 2010).

Each Q4PCR reaction was performed in a 10-µl total reaction volume containing 5 µl TaqMan Universal PCR Master Mix containing Rox (4304437; Applied Biosystems), 1 µl diluted genomic DNA, nuclease-free water, and the following primer and probe concentrations: PS, 675 nM of forward and reverse primers with 187.5 nM of PS internal probe; *env*, 90 nM of forward and reverse primers with 25 nM of *env* internal probe; *gag*, 337.5 nM of forward and reverse primers with 93.75 nM of *gag* internal probe; and *pol*, 675 nM of forward and reverse primers with 187.5 nM of *pol* internal probe. qPCR conditions were 94°C for 10 min, 40 cycles of 94°C for 15 s, and 60°C for 60 s. All qPCR reactions were performed in a 384-well plate format using the Applied Biosystem QuantStudio 6 Flex Real-Time PCR System.

### qPCR data analysis

We used QuantStudio Real-Time PCR Software version 1.3 (Thermo Fisher Scientific) for data analysis. The same baseline correction (start cycle, 3; end cycle, 10) and normalized reporter signal (ΔRn) threshold (ΔRn threshold = 0.025) was set manually for all targets/probes. Fluorescent signal above the threshold was used to determine the threshold cycle. Samples with a threshold cycle value between 10 and 40 of any probe or probe combination were identified. Samples showing reactivity with two or more of the four qPCR probes were selected for further processing.

### Nested NFL HIV-1 PCR (NFL2)

The nested PCR reaction (NFL2) was performed on undiluted 1-µl aliquots of the NFL1 PCR product. Reactions were performed in a 20-µl reaction volume using Platinum Taq High Fidelity polymerase (Thermo Fisher Scientific) and PCR primers 275F (5′-ACA​GGG​ACC​TGA​AAG​CGA​AAG-3′) and 280R (5′-CTA​GTT​ACC​AGA​GTC​ACA​CAA​CAG​ACG-3′; Ho et al., 2013) at a concentration of 800 nM. Touchdown cycling conditions were 94°C for 2 min and then 94°C for 30 s, 64°C for 30 s, and 68°C for 10 min for three cycles; 94°C for 30 s, 61°C for 30 s, and 68°C for 10 min for three cycles; 94°C for 30 s, 58°C for 30 s, and 68°C for 10 min for three cycles; 94°C for 30 s, 55°C for 30 s, and 68°C for 10 min for 41 cycles; and then 68°C for 10 min.

### Library preparation and sequencing

All nested PCR products from NFL2 were subjected to library preparation. The Qubit 3.0 Fluorometer and Qubit dsDNA BR Assay Kit (Thermo Fisher Scientific) were used to measure DNA concentrations. Samples were diluted to a concentration of 10–20 ng/µl. Tagmentation reactions were performed using 1 µl of diluted DNA, 0.25 µl Nextera TDE1 Tagment DNA enzyme (15027865), and 1.25 µl TD Tagment DNA buffer (15027866; Illumina). Tagmented DNA was ligated to unique i5/i7 barcoded primer combinations using the Illumina Nextera XT Index Kit v2 and KAPA HiFi HotStart ReadyMix (2X; KAPA Biosystems) and then purified using AmPure Beads XP (Agencourt). 384 purified samples were pooled into one library and then subjected to paired-end sequencing using Illumina MiSeq Nano 300 V2 cycle kits (Illumina) at a concentration of 12 pM.

### HIV-1 sequence assembly and annotation

HIV-1 sequence assembly was performed by our in-house pipeline (Defective and Intact HIV Genome Assembler), which is capable of reconstructing thousands of HIV genomes within hours via the assembly of raw sequencing reads into annotated HIV genomes. The steps executed by the pipeline are described briefly as follows. First, we removed PCR amplification and performed error correction using clumpify.sh from BBtools package v38.72 (https://sourceforge.net/projects/bbmap/). A quality control check was performed with Trim Galore package v0.6.4 (https://github.com/FelixKrueger/TrimGalore) to trim Illumina adapters and low-quality bases. We also used bbduk.sh from BBtools package to remove possible contaminant reads using HIV genome sequences, obtained from Los Alamos HIV database, as a positive control. We used a k-mer–based assembler, SPAdes v3.13.1, to reconstruct the HIV-1 sequences. The longest assembled contig was aligned via BLAST to a database of HIV genome sequences, obtained from Los Alamos, to set the correct orientation. Finally, the HIV genome sequence was annotated by aligning against Hxb2 using BLAST. Sequences with double peaks, i.e., regions indicating the presence of two or more viruses in the sample (cutoff consensus identity for any residue <70%), or samples with a limited number of reads (empty wells ≤500 sequencing reads) were omitted from downstream analyses. In the end, sequences were classified as intact or defective. Defective sequences were subdivided into more specific classifications according to their sequence structure: Major Splice Donor (MSD) Mutation, Non-functional, or Missing Internal Genes.

### Clone analysis for intact and defective sequences

Clones were defined by aligning sequences of each classification (Intact, MSD Mutation, Non-functional, and Missing Internal Genes) to HXB2 and calculating their Hamming distance. Sequences having a maximum of three differences between the first nucleotide of gag and the last nucleotide of nef (reference: HXB2) were considered members of the same clone if found more than once across sequences from baseline or all sorted populations (see Cell sorting) retrieved from each participant.

### Data availability

All data are available via National Center for Biotechnology Information GenBank under accession nos. MN090896–MN090943 and MT189273–MT191225.

### Online supplemental material

Fig. S1 shows the AIM assay flow cytometry plots from all participants. Fig. S2 shows the CFSE proliferation plots after 18 h of stimulation in the AIM assay. Table S1 shows the participants’ demographics. Table S2 shows the HLA typing of the study participants. Table S3 shows the gag limiting dilution calculations used for the Q4PCR assay. Table S4 shows the list of antibodies used in flow cytometry for the AIM assay.

## Supplementary Material

Table S1 shows donor characteristics.

Table S2 shows donor HLA genotyping.

Table S3 shows HIV-1 gag DNA enrichment in the different sorted populations.

Table S4 lists antibodies used in flow cytometry for cell sorting and identification of antigen-reactive cells.

## References

[bib1] Bailey J.R., Sedaghat A.R., Kieffer T., Brennan T., Lee P.K., Wind-Rotolo M., Haggerty C.M., Kamireddi A.R., Liu Y., Lee J., 2006 Residual human immunodeficiency virus type 1 viremia in some patients on antiretroviral therapy is dominated by a small number of invariant clones rarely found in circulating CD4+ T cells. J. Virol. 80:6441–6457. 10.1128/JVI.00591-06 16775332 PMC1488985

[bib2] Bruner K.M., and Cohn L.B. 2019 HIV-1 reservoir dynamics in CD4+ T cells. Curr. Opin. HIV AIDS. 14:108–114. 10.1097/COH.0000000000000521 30531293

[bib3] Bruner K., and Siliciano R. 2016. Compositions and methods related to characterizing proviral reservoirs. US patent US20180214473A1, filed by Johns Hopkins University April 22, 2016, pending.

[bib4] Bruner K.M., Murray A.J., Pollack R.A., Soliman M.G., Laskey S.B., Capoferri A.A., Lai J., Strain M.C., Lada S.M., Hoh R., 2016 Defective proviruses rapidly accumulate during acute HIV-1 infection. Nat. Med. 22:1043–1049. 10.1038/nm.4156 27500724 PMC5014606

[bib5] Bui J.K., Sobolewski M.D., Keele B.F., Spindler J., Musick A., Wiegand A., Luke B.T., Shao W., Hughes S.H., Coffin J.M., 2017 Proviruses with identical sequences comprise a large fraction of the replication-competent HIV reservoir. PLoS Pathog. 13:e1006283 10.1371/journal.ppat.1006283 28328934 PMC5378418

[bib6] Christensen-Quick A., Chaillon A., Yek C., Zanini F., Jordan P., Ignacio C., Caballero G., Gianella S., and Smith D. 2018 Influenza Vaccination Can Broadly Activate the HIV Reservoir During Antiretroviral Therapy. J. Acquir. Immune Defic. Syndr. 79:e104–e107. 10.1097/QAI.0000000000001829 30085954 PMC6185804

[bib7] Cohen Y.Z., Lorenzi J.C.C., Krassnig L., Barton J.P., Burke L., Pai J., Lu C.-L., Mendoza P., Oliveira T.Y., Sleckman C., 2018 Relationship between latent and rebound viruses in a clinical trial of anti-HIV-1 antibody 3BNC117. J. Exp. Med. 215:2311–2324. 10.1084/jem.20180936 30072495 PMC6122972

[bib8] Cohn L.B., da Silva I.T., Valieris R., Huang A.S., Lorenzi J.C.C., Cohen Y.Z., Pai J.A., Butler A.L., Caskey M., Jankovic M., and Nussenzweig M.C. 2018 Clonal CD4^+^ T cells in the HIV-1 latent reservoir display a distinct gene profile upon reactivation. Nat. Med. 24:604–609. 10.1038/s41591-018-0017-7 29686423 PMC5972543

[bib9] Cohn L.B., Silva I.T., Oliveira T.Y., Rosales R.A., Parrish E.H., Learn G.H., Hahn B.H., Czartoski J.L., McElrath M.J., Lehmann C., 2015 HIV-1 integration landscape during latent and active infection. Cell. 160:420–432. 10.1016/j.cell.2015.01.020 25635456 PMC4371550

[bib10] Craigie R., and Bushman F.D. 2012 HIV DNA integration. Cold Spring Harb. Perspect. Med. 2:a006890 10.1101/cshperspect.a006890 22762018 PMC3385939

[bib11] Crooks A.M., Bateson R., Cope A.B., Dahl N.P., Griggs M.K., Kuruc J.D., Gay C.L., Eron J.J., Margolis D.M., Bosch R.J., and Archin N.M. 2015 Precise Quantitation of the Latent HIV-1 Reservoir: Implications for Eradication Strategies. J. Infect. Dis. 212:1361–1365. 10.1093/infdis/jiv218 25877550 PMC4601910

[bib12] Dan J.M., Lindestam Arlehamn C.S., Weiskopf D., da Silva Antunes R., Havenar-Daughton C., Reiss S.M., Brigger M., Bothwell M., Sette A., and Crotty S. 2016 A Cytokine-Independent Approach To Identify Antigen-Specific Human Germinal Center T Follicular Helper Cells and Rare Antigen-Specific CD4+ T Cells in Blood. J. Immunol. 197:983–993. 10.4049/jimmunol.1600318 27342848 PMC4955771

[bib13] Demoustier A., Gubler B., Lambotte O., de Goër M.-G., Wallon C., Goujard C., Delfraissy J.-F., and Taoufik Y. 2002 In patients on prolonged HAART, a significant pool of HIV infected CD4 T cells are HIV-specific. AIDS. 16:1749–1754. 10.1097/00002030-200209060-00006 12218385

[bib14] Douek D.C., Brenchley J.M., Betts M.R., Ambrozak D.R., Hill B.J., Okamoto Y., Casazza J.P., Kuruppu J., Kunstman K., Wolinsky S., 2002 HIV preferentially infects HIV-specific CD4+ T cells. Nature. 417:95–98. 10.1038/417095a 11986671

[bib15] Einkauf K.B., Lee G.Q., Gao C., Sharaf R., Sun X., Hua S., Chen S.M.Y., Jiang C., Lian X., Chowdhury F.Z., 2019 Intact HIV-1 proviruses accumulate at distinct chromosomal positions during prolonged antiretroviral therapy. J. Clin. Invest. 129:988–998. 10.1172/JCI124291 30688658 PMC6391088

[bib16] Gaebler C., Lorenzi J.C.C., Oliveira T.Y., Nogueira L., Ramos V., Lu C.-L., Pai J.A., Mendoza P., Jankovic M., Caskey M., and Nussenzweig M.C. 2019 Combination of quadruplex qPCR and next-generation sequencing for qualitative and quantitative analysis of the HIV-1 latent reservoir. J. Exp. Med. 216:2253–2264. 10.1084/jem.20190896 31350309 PMC6781006

[bib17] Hatano H., Strain M.C., Scherzer R., Bacchetti P., Wentworth D., Hoh R., Martin J.N., McCune J.M., Neaton J.D., Tracy R.P., 2013 Increase in 2-long terminal repeat circles and decrease in D-dimer after raltegravir intensification in patients with treated HIV infection: a randomized, placebo-controlled trial. J. Infect. Dis. 208:1436–1442. 10.1093/infdis/jit453 23975885 PMC3789577

[bib18] Havenar-Daughton C., Reiss S.M., Carnathan D.G., Wu J.E., Kendric K., Torrents de la Peña A., Kasturi S.P., Dan J.M., Bothwell M., Sanders R.W., 2016 Cytokine-Independent Detection of Antigen-Specific Germinal Center T Follicular Helper Cells in Immunized Nonhuman Primates Using a Live Cell Activation-Induced Marker Technique. J. Immunol. 197:994–1002. 10.4049/jimmunol.1600320 27335502 PMC4955744

[bib19] Henrich T.J., Hobbs K.S., Hanhauser E., Scully E., Hogan L.E., Robles Y.P., Leadabrand K.S., Marty F.M., Palmer C.D., Jost S., 2017 Human Immunodeficiency Virus Type 1 Persistence Following Systemic Chemotherapy for Malignancy. J. Infect. Dis. 216:254–262. 10.1093/infdis/jix265 28838149 PMC5853412

[bib20] Hey-Nguyen W.J., Bailey M., Xu Y., Suzuki K., Van Bockel D., Finlayson R., Leigh Brown A., Carr A., Cooper D.A., Kelleher A.D., 2019 HIV-1 DNA Is Maintained in Antigen-Specific CD4+ T Cell Subsets in Patients on Long-Term Antiretroviral Therapy Regardless of Recurrent Antigen Exposure. AIDS Res. Hum. Retroviruses. 35:112–120. 10.1089/aid.2018.0235 30511878

[bib21] Hiener B., Horsburgh B.A., Eden J.-S., Barton K., Schlub T.E., Lee E., von Stockenstrom S., Odevall L., Milush J.M., Liegler T., 2017 Identification of Genetically Intact HIV-1 Proviruses in Specific CD4^+^ T Cells from Effectively Treated Participants. Cell Rep. 21:813–822. 10.1016/j.celrep.2017.09.081 29045846 PMC5960642

[bib22] Ho Y.-C., Shan L., Hosmane N.N., Wang J., Laskey S.B., Rosenbloom D.I.S., Lai J., Blankson J.N., Siliciano J.D., and Siliciano R.F. 2013 Replication-competent noninduced proviruses in the latent reservoir increase barrier to HIV-1 cure. Cell. 155:540–551. 10.1016/j.cell.2013.09.020 24243014 PMC3896327

[bib23] Hosmane N.N., Kwon K.J., Bruner K.M., Capoferri A.A., Beg S., Rosenbloom D.I.S., Keele B.F., Ho Y.-C., Siliciano J.D., and Siliciano R.F. 2017 Proliferation of latently infected CD4^+^ T cells carrying replication-competent HIV-1: Potential role in latent reservoir dynamics. J. Exp. Med. 214:959–972. 10.1084/jem.20170193 28341641 PMC5379987

[bib24] Jones R.B., Kovacs C., Chun T.-W., and Ostrowski M.A. 2012 Short communication: HIV type 1 accumulates in influenza-specific T cells in subjects receiving seasonal vaccination in the context of effective antiretroviral therapy. AIDS Res. Hum. Retroviruses. 28:1687–1692. 10.1089/aid.2012.0115 22734882 PMC3505056

[bib25] Kristoff J., Palma M.L., Garcia-Bates T.M., Shen C., Sluis-Cremer N., Gupta P., Rinaldo C.R., and Mailliard R.B. 2019 Type 1-programmed dendritic cells drive antigen-specific latency reversal and immune elimination of persistent HIV-1. EBioMedicine. 43:295–306. 10.1016/j.ebiom.2019.03.077 30952614 PMC6557749

[bib26] Kuo H.-H., Ahmad R., Lee G.Q., Gao C., Chen H.-R., Ouyang Z., Szucs M.J., Kim D., Tsibris A., Chun T.-W., 2018 Anti-apoptotic Protein BIRC5 Maintains Survival of HIV-1-Infected CD4^+^ T Cells. Immunity. 48:1183–1194.e5. 10.1016/j.immuni.2018.04.004 29802019 PMC6013384

[bib27] Li B., Gladden A.D., Altfeld M., Kaldor J.M., Cooper D.A., Kelleher A.D., and Allen T.M. 2007 Rapid reversion of sequence polymorphisms dominates early human immunodeficiency virus type 1 evolution. J. Virol. 81:193–201. 10.1128/JVI.01231-06 17065207 PMC1797245

[bib28] Llewelyn M., Sriskandan S., Terrazzini N., Cohen J., and Altmann D.M. 2006 The TCR Vbeta signature of bacterial superantigens spreads with stimulus strength. Int. Immunol. 18:1433–1441. 10.1093/intimm/dxl076 16893924

[bib29] Lorenzi J.C.C., Cohen Y.Z., Cohn L.B., Kreider E.F., Barton J.P., Learn G.H., Oliveira T., Lavine C.L., Horwitz J.A., Settler A., 2016 Paired quantitative and qualitative assessment of the replication-competent HIV-1 reservoir and comparison with integrated proviral DNA. Proc. Natl. Acad. Sci. USA. 113:E7908–E7916. 10.1073/pnas.1617789113 27872306 PMC5150408

[bib30] Lu C.-L., Pai J.A., Nogueira L., Mendoza P., Gruell H., Oliveira T.Y., Barton J., Lorenzi J.C.C., Cohen Y.Z., Cohn L.B., 2018 Relationship between intact HIV-1 proviruses in circulating CD4^+^ T cells and rebound viruses emerging during treatment interruption. Proc. Natl. Acad. Sci. USA. 115:E11341–E11348. 10.1073/pnas.1813512115 30420517 PMC6275529

[bib31] Maldarelli F., Wu X., Su L., Simonetti F.R., Shao W., Hill S., Spindler J., Ferris A.L., Mellors J.W., Kearney M.F., 2014 HIV latency. Specific HIV integration sites are linked to clonal expansion and persistence of infected cells. Science. 345:179–183. 10.1126/science.1254194 24968937 PMC4262401

[bib32] McManus W.R., Bale M.J., Spindler J., Wiegand A., Musick A., Patro S.C., Sobolewski M.D., Musick V.K., Anderson E.M., Cyktor J.C., 2019 HIV-1 in lymph nodes is maintained by cellular proliferation during antiretroviral therapy. J. Clin. Invest. 130:4629–4642. 10.1172/JCI126714 PMC6819093 31361603

[bib33] Mendoza P., Gruell H., Nogueira L., Pai J.A., Butler A.L., Millard K., Lehmann C., Suárez I., Oliveira T.Y., Lorenzi J.C.C., 2018 Combination therapy with anti-HIV-1 antibodies maintains viral suppression. Nature. 561:479–484. 10.1038/s41586-018-0531-2 30258136 PMC6166473

[bib34] Niessl J., Baxter A.E., Mendoza P., Jankovic M., Cohen Y.Z., Butler A.L., Lu C.-L., Dubé M., Shimeliovich I., Gruell H., 2020 Combination anti-HIV-1 antibody therapy is associated with increased virus-specific T cell immunity. Nat. Med. 26:222–227. 10.1038/s41591-019-0747-1 32015556 PMC7018622

[bib35] Palmer S., Wiegand A.P., Maldarelli F., Bazmi H., Mican J.M., Polis M., Dewar R.L., Planta A., Liu S., Metcalf J.A., 2003 New real-time reverse transcriptase-initiated PCR assay with single-copy sensitivity for human immunodeficiency virus type 1 RNA in plasma. J. Clin. Microbiol. 41:4531–4536. 10.1128/JCM.41.10.4531-4536.2003 14532178 PMC254331

[bib36] Pinzone M.R., VanBelzen D.J., Weissman S., Bertuccio M.P., Cannon L., Venanzi-Rullo E., Migueles S., Jones R.B., Mota T., Joseph S.B., 2019 Longitudinal HIV sequencing reveals reservoir expression leading to decay which is obscured by clonal expansion. Nat. Commun. 10:728 10.1038/s41467-019-08431-7 30760706 PMC6374386

[bib37] Reeves D.B., Duke E.R., Wagner T.A., Palmer S.E., Spivak A.M., and Schiffer J.T. 2018 A majority of HIV persistence during antiretroviral therapy is due to infected cell proliferation. Nat. Commun. 9:4811 10.1038/s41467-018-06843-5 30446650 PMC6240116

[bib38] Reiss S., Baxter A.E., Cirelli K.M., Dan J.M., Morou A., Daigneault A., Brassard N., Silvestri G., Routy J.-P., Havenar-Daughton C., 2017 Comparative analysis of activation induced marker (AIM) assays for sensitive identification of antigen-specific CD4 T cells. PLoS One. 12:e0186998 10.1371/journal.pone.0186998 29065175 PMC5655442

[bib39] Schmid A., Gianella S., von Wyl V., Metzner K.J., Scherrer A.U., Niederöst B., Althaus C.F., Rieder P., Grube C., Joos B., 2010 Profound depletion of HIV-1 transcription in patients initiating antiretroviral therapy during acute infection. PLoS One. 5:e13310 10.1371/journal.pone.0013310 20967271 PMC2953504

[bib40] Schröder A.R.W., Shinn P., Chen H., Berry C., Ecker J.R., and Bushman F. 2002 HIV-1 integration in the human genome favors active genes and local hotspots. Cell. 110:521–529. 10.1016/S0092-8674(02)00864-4 12202041

[bib41] Sengupta S., and Siliciano R.F. 2018 Targeting the Latent Reservoir for HIV-1. Immunity. 48:872–895. 10.1016/j.immuni.2018.04.030 29768175 PMC6196732

[bib42] Siliciano J.D., Kajdas J., Finzi D., Quinn T.C., Chadwick K., Margolick J.B., Kovacs C., Gange S.J., and Siliciano R.F. 2003 Long-term follow-up studies confirm the stability of the latent reservoir for HIV-1 in resting CD4+ T cells. Nat. Med. 9:727–728. 10.1038/nm880 12754504

[bib50] van Sighem A., Zhang S., Reiss P., Gras L., van der Ende M., Kroon F., Prins J., and de Wolf F. 2008 Immunologic, virologic, and clinical consequences of episodes of transient viremia during suppressive combination antiretroviral therapy. J. Acquir. Immune Defic. Syndr. 48(1):104–108. 10.1097/QAI.0b013e31816a1d4f 18285709

[bib43] Siliciano R.F., and Greene W.C. 2011 HIV latency. Cold Spring Harb. Perspect. Med. 1:a007096 10.1101/cshperspect.a007096 22229121 PMC3234450

[bib44] Simonetti F.R., Sobolewski M.D., Fyne E., Shao W., Spindler J., Hattori J., Anderson E.M., Watters S.A., Hill S., Wu X., 2016 Clonally expanded CD4+ T cells can produce infectious HIV-1 in vivo. Proc. Natl. Acad. Sci. USA. 113:1883–1888. 10.1073/pnas.1522675113 26858442 PMC4763755

[bib45] Stanley S.K., Ostrowski M.A., Justement J.S., Gantt K., Hedayati S., Mannix M., Roche K., Schwartzentruber D.J., Fox C.H., and Fauci A.S. 1996 Effect of immunization with a common recall antigen on viral expression in patients infected with human immunodeficiency virus type 1. N. Engl. J. Med. 334:1222–1230. 10.1056/NEJM199605093341903 8606717

[bib46] Tobin N.H., Learn G.H., Holte S.E., Wang Y., Melvin A.J., McKernan J.L., Pawluk D.M., Mohan K.M., Lewis P.F., Mullins J.I., and Frenkel L.M. 2005 Evidence that low-level viremias during effective highly active antiretroviral therapy result from two processes: expression of archival virus and replication of virus. J. Virol. 79:9625–9634. 10.1128/JVI.79.15.9625-9634.2005 16014925 PMC1181593

[bib47] Wagner T.A., McLaughlin S., Garg K., Cheung C.Y.K., Larsen B.B., Styrchak S., Huang H.C., Edlefsen P.T., Mullins J.I., and Frenkel L.M. 2014 HIV latency. Proliferation of cells with HIV integrated into cancer genes contributes to persistent infection. Science. 345:570–573. 10.1126/science.1256304 25011556 PMC4230336

[bib48] Wang Z., Gurule E.E., Brennan T.P., Gerold J.M., Kwon K.J., Hosmane N.N., Kumar M.R., Beg S.A., Capoferri A.A., Ray S.C., 2018 Expanded cellular clones carrying replication-competent HIV-1 persist, wax, and wane. Proc. Natl. Acad. Sci. USA. 115:E2575–E2584. 10.1073/pnas.1720665115 29483265 PMC5856552

[bib49] Wiegand A., Spindler J., Hong F.F., Shao W., Cyktor J.C., Cillo A.R., Halvas E.K., Coffin J.M., Mellors J.W., and Kearney M.F. 2017 Single-cell analysis of HIV-1 transcriptional activity reveals expression of proviruses in expanded clones during ART. Proc. Natl. Acad. Sci. USA. 114:E3659–E3668. 10.1073/pnas.1617961114 28416661 PMC5422779

