# Supplementary figures and images for: Antigen-responsive CD4+ T cell clones contribute to the HIV-1 latent reservoir
Source: J Exp Med. 2020 Apr 20;217(7):e20200051. doi: 10.1084/jem.20200051 (PMC7336300; doi:10.1084/jem.20200051)

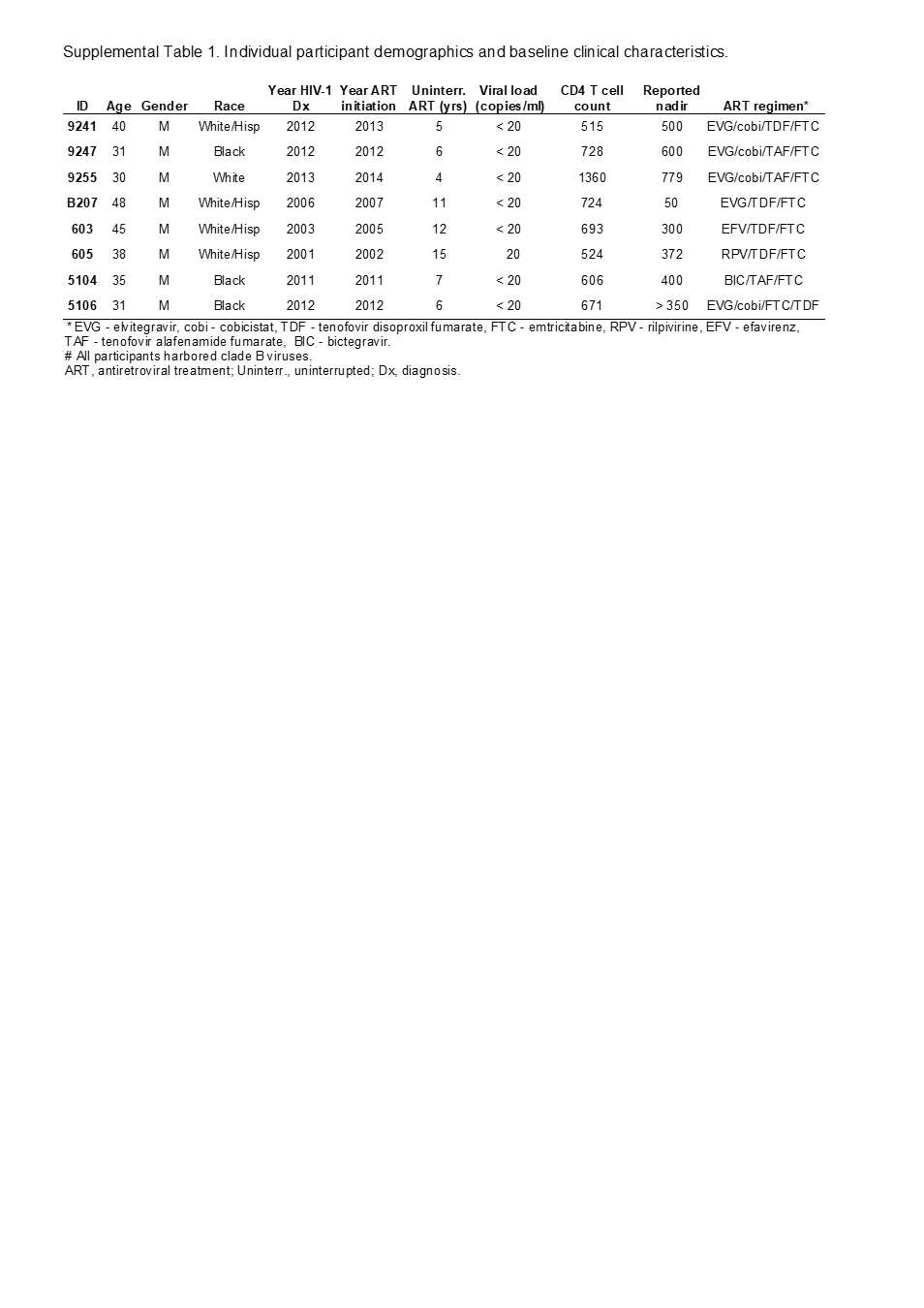

Supplement: Table S1 — shows donor characteristics. [file JEM_20200051_TableS1.docx]
