# Supplementary material for: Antigen-responsive CD4+ T cell clones contribute to the HIV-1 latent reservoir
Source: J Exp Med. 2020 Apr 20;217(7):e20200051. doi: 10.1084/jem.20200051 (PMC7336300; doi:10.1084/jem.20200051)
Supplement: Table S3 — shows HIV-1 gag DNA enrichment in the different sorted populations. [file JEM_20200051_TableS3.docx]

HIV-1 gag DNA was detected by qPCR with a gag probe testing three cellular DNA dilutions, based on previous Q4PCR analysis of these participants. A total of 16 wells for each dilution was analyzed, and the number of cells per well was determined based on cellular DNA concentration. Final cell concentration for Q4PCR in each sample was determined by the dilution with less than 30% HIV-1 gag positive wells to achieve a single virus dilution.
