# Supplementary material for: Antigen-responsive CD4+ T cell clones contribute to the HIV-1 latent reservoir
Source: J Exp Med. 2020 Apr 20;217(7):e20200051. doi: 10.1084/jem.20200051 (PMC7336300; doi:10.1084/jem.20200051)
Supplement: Supplementary file 3 [file JEM_20200051_TableS2.docx]

Supplemental Table 2. HLA typing of study participants.

	HLA Class I						HLA Class II															
**ID**	**HLA-A**	**HLA-A**	**HLA-B**	**HLA-B**	**HLA-C**	**HLA-C**	**DRB1**	**DRB1**	**DRB3**	**DRB3**	**DRB4**	**DRB4**	**DRB5**	**DRB5**	**DQB1**	**DQB1**	**DPB1**	**DPB1**	**DQA1**	**DQA1**	**DPA1**	**DPA1**
**9241**	23:01	33:01	14:02	44:03	04:01	08:02	N/A	N/A	N/A	N/A	N/A	N/A	N/A	N/A	N/A	N/A	N/A	N/A	N/A	N/A	N/A	N/A
**9247**	33:01	34:02	44:03	78:01	04:01	16:01	N/A	N/A	N/A	N/A	N/A	N/A	N/A	N/A	N/A	N/A	N/A	N/A	N/A	N/A	N/A	N/A
**9255**	03:01	25:01	18:01	44:27	07:04	12:03	N/A	N/A	N/A	N/A	N/A	N/A	N/A	N/A	N/A	N/A	N/A	N/A	N/A	N/A	N/A	N/A
**B207**	02:17	23:01	07:02	35:03	07:02	14:02	14:54	16:01	02:02	-	-	-	01:01	-	05:02	05:03	04:01	09:01	01:02	01:04	01:03	02:01
**603**	02:01	33:01	44:03	51:01	02:02	02:02	04:11	08:01	-	-	01:03	-	-	-	03:01	04:02	04:01	04:02	03:01	04:01	01:03	01:03
**605**	02:01	03:01	39:05	58:01	03:02	07:02	04:11	13:04	02:02	-	01:03	-	-	-	03:02	03:19	04:02	131:01	03:01	05:05	01:03	02:01
**5104**	02:01	74:01	57:03	78:01	16:01	18:02	01:02	16:02	-	-	-	-	02:21	-	05:01	05:02	04:01	85:01	01:01	01:02	02:12	03:01
**5106**	03:01	03:01	38:01	57:03	12:03	18:02	07:01	13:01	01:01	-	01:03	-	-	-	02:02	06:03	02:01	105:01	01:10	02:01	01:03	03:01

N/A: not available
