# Supplementary material for: Antigen-responsive CD4+ T cell clones contribute to the HIV-1 latent reservoir
Source: J Exp Med. 2020 Apr 20;217(7):e20200051. doi: 10.1084/jem.20200051 (PMC7336300; doi:10.1084/jem.20200051)
Supplement: Supplementary file 4 [file JEM_20200051_TableS4.docx]

Supplemental Table 4. List of antibodies used for flow cytometry.

**Marker**	**Fluorochrome**	**Clone**	**Supplier**	**Cat. No.**	**Dilution**
CD8	BV711	RPA-T8	Biolegend	301044	1/50
PD-L1	BV421	29E2A3	Biolegend	329714	1/20
4-1BB	PE-Cy7	4B4-1	Biolegend	309818	1/20
CD14	BV510	M5E2	Biolegend	301842	1/33
CD19	BV510	H1B19	Biolegend	302242	1/33
CD4	APC	RPA-T4	Biolegend	300514	1/133
CD69	BV650	FN50	Biolegend	310934	1/28
